# An Obligatory Role of NF-κB in Mediating Bone Marrow Derived Endothelial Progenitor Cell Recruitment and Proliferation Following Endotoxemic Multiple Organ Injury in Mice

**DOI:** 10.1371/journal.pone.0111087

**Published:** 2014-10-21

**Authors:** Sun-Zhong Mao, Xiaobing Ye, Gang Liu, Dongmei Song, Shu Fang Liu

**Affiliations:** 1 Centers for Heart and Lung Research and Pulmonary and Critical Care Medicine, the Feinstein Institute for Medical Research, Manhasset, New York, United States of America; 2 Institute of Hypoxia Medicine, Wenzhou Medical University, Wenzhou, Zhejiang, China; University of Kentucky, United States of America

## Abstract

**Background:**

Recruitment of bone marrow derived endothelial progenitor cells (BMDEPCs) alleviates multiple organ injury (MOI) and improves outcomes. However, mechanisms mediating BMDEPC recruitment following septic MOI remain largely unknown. This study characterized the kinetics of BMDEPC recruitment and proliferation and defined the role of NF-κB in regulating BMDEPC recruitment and proliferation.

**Methods and Main Findings:**

Chimeric mice with an intact or disrupted NF-κB p50 gene and BMDEPC-restricted expression of green fluorescent protein were created and injected with LPS (2 mg/kg, i.p.). BMDEPC recruitment and proliferation in multiple organs were quantified. BMDEPC recruitment and proliferation are highly organ-dependent. Lungs had the highest number of BMDEPC recruitment, whereas heart, liver and kidney had only a small fraction of the number of BMDEPCs in lungs. Number of proliferating BMDEPCs was several-fold higher in lungs than in other 3 organs. Kinetically, BMDEPC recruitment into different organs showed different time course profiles. NF-κB plays obligatory roles in mediating BMDEPC recruitment and proliferation. Universal deletion of NF-κB p50 gene inhibited LPS-induced BMDEPC recruitment and proliferation by 95% and 69% in heart. However, the contribution of NF-κB to these regulations varies significantly between organs. In liver, universal p50 gene deletion reduced LPS-induced BMDEPC recruitment and proliferation only by 49% and 35%. NF-κB activities in different tissue compartments play distinct roles. Selective p50 gene deletion either in stromal/parenchymal cells or in BM/blood cells inhibited BMDEPC recruitment by a similar extent. However, selective p50 gene deletion in BM/blood cells inhibited, but in stromal/parenchymal cells augmented BMDEPC proliferation.

**Conclusions:**

BMDEPC recruitment and proliferation display different kinetics in different organs following endotoxemic MOI. NF-κB plays obligatory and organ-dependent roles in regulating BMDEPC recruitment and proliferation. NF-κB activities in different tissue compartments play distinct roles in regulating BMDEPC proliferation.

## Introduction

Multiple organ injury (MOI) is associated with remarkably increased mobilization and recruitment of endothelial progenitor cells (EPCs) and other stem/progenitor cells (SPCs) from bone marrow (BM). Although whether and how BM-derived EPCs/SPCs contribute to organ repair are subjects of intense debate [1]–[6], it is generally agreed that an increased recruitment of BM-derived EPCs/SPCs, either from mobilization of endogenous EPCs/SPCs or by administration of exogenous EPCs/SPCs, has beneficial effects [5]–[24]. Clinical studies showed that patients with organ injury had increased numbers of circulating EPCs, which correlated with better prognosis and survival rate [7]–[9]. Animal experiments demonstrated that exogenously administered EPCs or other SPCs blunted systemic and organ inflammation, alleviated organ damage [10]–[13], reduced endothelial permeability [14] and improved survival [11], [14], [15]. The strong anti-injury and organ protective effects of EPCs/SPCs suggest that enhancing EPCs/SPCs recruitment could be a good strategy for treating MOI. In this regard, elucidation of mechanisms and pathways regulating EPC/SPC recruitment and proliferation in injured organ may provide important information guiding the development of such a therapy. However, studies characterizing the kinetics of EPC recruitment and proliferation following septic MOI are rare. Mechanisms and pathways regulating EPC recruitment following septic MOI remain largely to be elucidated, although there is limited information available in mechanical arterial injury and atherosclerotic renal artery stenosis models [25], [26].

Recent studies showed that EPCs/SPCs conferred protection against organ injury and improved outcomes by paracrine mechanisms: by secreting immunomodulating mediators [15]–[18] and/or angiogenic factors [19], [20], and by transferring mitochondria, protein or microRNA to resident cells in injured organ via the release of microvesicles/exosomes [21], [22], or via direct cell-cell interactions [23], [24]. Thus, communications between BM-derived EPCs (BMDEPCs) and stromal/parenchymal cells could be critical for BMDEPC-mediated anti-injury actions. However, little is known about the mechanisms and pathways regulating the intercellular communications. In this regard, deciphering the role of individual compartment-specific signaling in the regulation of EPC recruitment and function may shed some light. No such a study has been reported.

NF-κB is a multi-faceted transcription factor that plays important roles in many cellular, physiological and pathological processes [27]–[29]. NF-κB is a key mediator of immune and inflammatory responses [29], but also plays a role in inflammation resolution [30]. The antiapoptotic and proliferative actions of NF-κB in parenchymal cells may contribute to organ repair [31], whereas this NF-κB function in macrophages and other inflammatory cells promotes inflammation [27], [29]. NF-κB regulates tissue homeostasis [32] and energy balance [33]. Although the contributions of NF-κB to organ inflammation and injury have been extensively studied [29], [33]–[37], the potential role of NF-κB in organ repair is less clear. Since MOI is associated with remarkably increased BMDEPC recruitment, NF-κB may play a role in regulating BMDEPC recruitment and proliferation following MOI. This potential function of NF-κB has not been studied.

This study characterized the kinetics of BMDEPC recruitment and proliferation, defined the role of NF-κB in regulating BMDEPC recruitment and proliferation, and deciphered the role of compartment-specific NF-κB activity in this regulation following endotoxemic MOI. We created several new chimeric mouse strains with universal or cell type-restricted deletion of NF-κB p50 gene and BMDEPC-restricted overexpression of green fluorescent protein (GFP). Using these mouse models, we demonstrated that BMDEPC recruitment and proliferation displayed different kinetics in different organs following endotoxemic MOI. NF-κB played obligatory and organ-dependent roles in mediating BMDEPC recruitment and proliferation. NF-κB activity in different tissue compartment played distinct role in regulating BMDEPC proliferation. Our data provides important new insights into the mechanisms regulating BMDEPC recruitment and proliferation following endotoxemic MOI.

## Materials and Methods

### Animal models

All animal experiments were approved by Institutional Animal Care and Use Committee of the Feinstein Institute for Medical Research (Number, 2012-033), and were carried out in strict accordance with the recommendations in the Guide for the Care and Use of Laboratory Animals of the National Institutes of Health. Mice deficient in NF-κB p50 gene (p50-KO) and transgenic mice overexpressing GFP on endothelial lineage cells (Tie2-GFP) were purchased from Jackson Laboratory (Bar Harbor, ME). The p50-KO mice were on C57BL/6 genetic background and were backcrossed to FVB genetic background for at least 6 generations. We initially created a new NG (p50-KO-GFP) mouse strain by crossbreeding between p50-KO and Tie2-GFP mice. The NG mice are deficient in NF-κB p50 gene in all cell types and express GFP on endothelial lineage cells. We then created WT-GFP-BM, p50-GFP-BM, WT-NG-BM or p50-NG-BM chimeric mouse strain by transplanting lethally irradiated wild type (WT), p50-KO, WT or p50-KO mice with BMs from the Tie-GFP or NG mice. The p50-GFP-BM, WT-NG-BM or p50-NG-BM chimeras are deficient in NF-κB p50 gene in stromal/parenchymal cells, in BM/blood cells or in all cell types. All 4 chimeric mouse strains overexpress GFP in BMDEPCs in a cell-restricted manner. A description of the mouse strains used for this study was given in Table S1.

### Bone marrow transplant

Recipient mice (6–8 weeks) received a lethal dose of whole body X-ray irradiation (900 Rads) and transplanted with donor BM cells. Donor mice were euthanized. The femurs and tibias were removed aseptically and marrow cavities flushed with Ca++, Mg++-free Hanks' Balanced Salt Solution. Single cell suspension was prepared, washed twice, counted, and resuspended in sterile RPMI medium at 3×10^7^cells/ml prior to transplantation. Each irradiated recipient was injected with 1×10^7^ BM cells through tail vein.

Two months after BM transplantation, the chimeras were used for experiments or tissue collection. At the completion of experiment, mice were euthanized and BM collected. Reconstitution of recipient BM with donor BM cells was confirmed and degree of donor BM chimerism analyzed by fluorescence activated cell sorting.

### Fluorescence activated cell sorting (FACS)

To evaluate BM chimerism, BM mononuclear cells were isolated from donors, recipients and chimeras using Ficoll density gradient centrifugation, and stained with PE/Cy7-anti-CD31 (BD Biosciences, San Jose, CA) plus Alexa Fluor 488-anti-GFP (Life Technologies, Grand Island, NY) antibodies. Fluorescence minus one controls were stained in parallel in all analyses. Cells were analyzed using FACS to determine percentage of GFP+ EPCs in BM mononuclear cell population. Degree of donor chimerism was determined by comparing percentage of GFP+ EPCs between each chimera and its respective donor.

### Assessment of LPS-induced MOI

Control or LPS group of WT mice were injected with saline (1 ml/kg, i.p.) or *Escherichia coli* LPS (0111:B4, 2 mg/kg, i.p.). Organ inflammation and injury were assessed 24 hours after LPS injection by histological examination, by measuring tissue level of myeloperoxidase (MPO) activity (as an indicator of neutrophil infiltration), as we have previously described [34], and by measuring tissue level of IL-6 using IL-6 ELISA kits (eBioscience, San Diego, CA).

### Quantification of recruited and proliferating BMDEPCs

Two months after BM transplantation, the chimeric mice were injected with saline (controls) or LPS (2 mg/kg, i.p.). The *in situ* cell proliferation kit (Roche Diagnostics, Branchburg, NJ) was used for *in situ* detection of proliferating BMDEPCs. To label proliferating cells *in vivo*, mice were injected with bromodeoxyuridine (BrdU, 100 μg/kg, i.v.) 4 hours prior to tissue collection.

For studying time course of BMDEPC recruitment and proliferation, cryosections of heart, lungs, liver and kidney were prepared from control mice and mice 12, 24, 48, 96 or 144 hours after LPS injection, fixed with paraformaldehyde, permeabilized, stained with anti-GFP (genetic marker for BMDEPCs) antibody or stained with anti-GFP plus anti-BrdU (marker for proliferating cells) antibodies (Abcam, Cambridge, MA), followed by Alexa Fluor 594 conjugated or/and Alexa Fluor 488 conjugated secondary antibody (Invitrogen, Carlsbad, CA). Nuclei were counterstained with 4′, 6-diamidino-2-phenylindole (DAPI). Numbers of GFP+ cells (BMDEPCs) or GFP+/BrdU+ cells (proliferating BMDEPCs) were counted and expressed as a percentage of total cells as revealed by DAPI nuclear staining.

To examine the effects of NF-κB p50 gene deletion on BMDEPC recruitment and proliferation, the numbers of GFP+ BMDEPCs or GFP+/BrdU+ proliferating BMDEPCs in the 4 organs were compared between WT-GFP-BM and p50-NG-BM, p50-GFP-BM or WT-NG-BM chimeric mice.

### Electrophoretic mobility shift assay (EMSA)

To confirm that p50 gene deletion in p50-NG-BM chimera blocks LPS-induced NF-κB activation, we compared LPS-induced NF-κB binding activity between WT-GFP-BM and p50-NG-BM mice. Nuclear proteins were extracted from heart, lungs, liver and kidney. NF-κB DNA binding activity was measured by EMSA using ^32^P-labeled NF-κB consensus oligonucleotide as we have previously described [37]. The specificity of NF-κB DNA binding was confirmed in competition reactions in which 50-fold molar excess unlabelled NF-κB oligonucleotide was added to the binding reaction 10 minutes prior to the addition of labeled probe.

### Statistical analysis

Data were expressed as mean ± SEM and analyzed using ANOVA or Kruskal-Wallis rank test followed by Holm-Sidak method or Student-Newman-Keuls Method for *post hoc* analysis. The null hypothesis was rejected at 5% level.

## Results

### Characterization of BMDEPC recruitment and proliferation following endotoxemic MOI

To better understand the mechanisms regulating BMDEPC recruitment and proliferation, we first characterized the profiles of BMDEPC recruitment and proliferation in multiple organs following endotoxemic MOI. The p50-NG-BM mice are extremely sensitive to LPS. We found that LPS at 5 mg/kg (i.p.) produced a mortality rate of ∼95% by 24 hours in p50-NG-BM mice. This study used low dose LPS (2 mg/kg, i.p.), which has been previously shown to induce organ injury in lung [38], kidney [39] and liver [40]. Here, we demonstrated that this low dose of LPS caused inflammation, neutrophil infiltration and histological evidence of tissue injury in lungs, liver and kidney (Fig. S1), confirming that this dose of LPS causes MOI.

We generated WT-GFP-BM chimeras and confirmed high level of BM chimerism by demonstrating that 95% of BM cells in the chimeras were donor BM origin (Fig. S2A). Tie2 is reported to be expressed on a subset of hematopoietic cells [41]. However, a phenotypic characterization of lung cells from the Tie2-GFP mice demonstrated that 89.5% of all GFP+ cells are CD45−/CD31+ endothelial lineage cells, and only 0.4% of them are CD45+/CD31+ hematopoietic cells [3]. Since CD31+ BM-derived hematopoietic progenitor cells have strong angiogenic and vasculogenic capacities, and high ability to give rise to new endothelial cells [42], the CD31+ BM-derived hematopoietic progenitor cells and BMDEPCs are likely to play similar roles in alleviating organ injury or/and in promoting organ repair. It is, therefore, not necessary to differentiate one from another in this study. We referred all BM-derived GFP+ cells as BMDEPCs, which would include all BMDEPCs and a small portion (∼0.5%) of BM-derived hematopoietic progenitor cells.

Two months after BM transplantation, mice were injected with saline or LPS, and then with bromodeoxyuridine (BrdU) 4 hours prior to tissue collection to label proliferating cells *in vivo*. BMDEPCs recruited into or proliferating in heart, lungs, liver and kidney were identified by GFP staining or BrdU plus GFP double staining, and counted.

**Figure 1:**
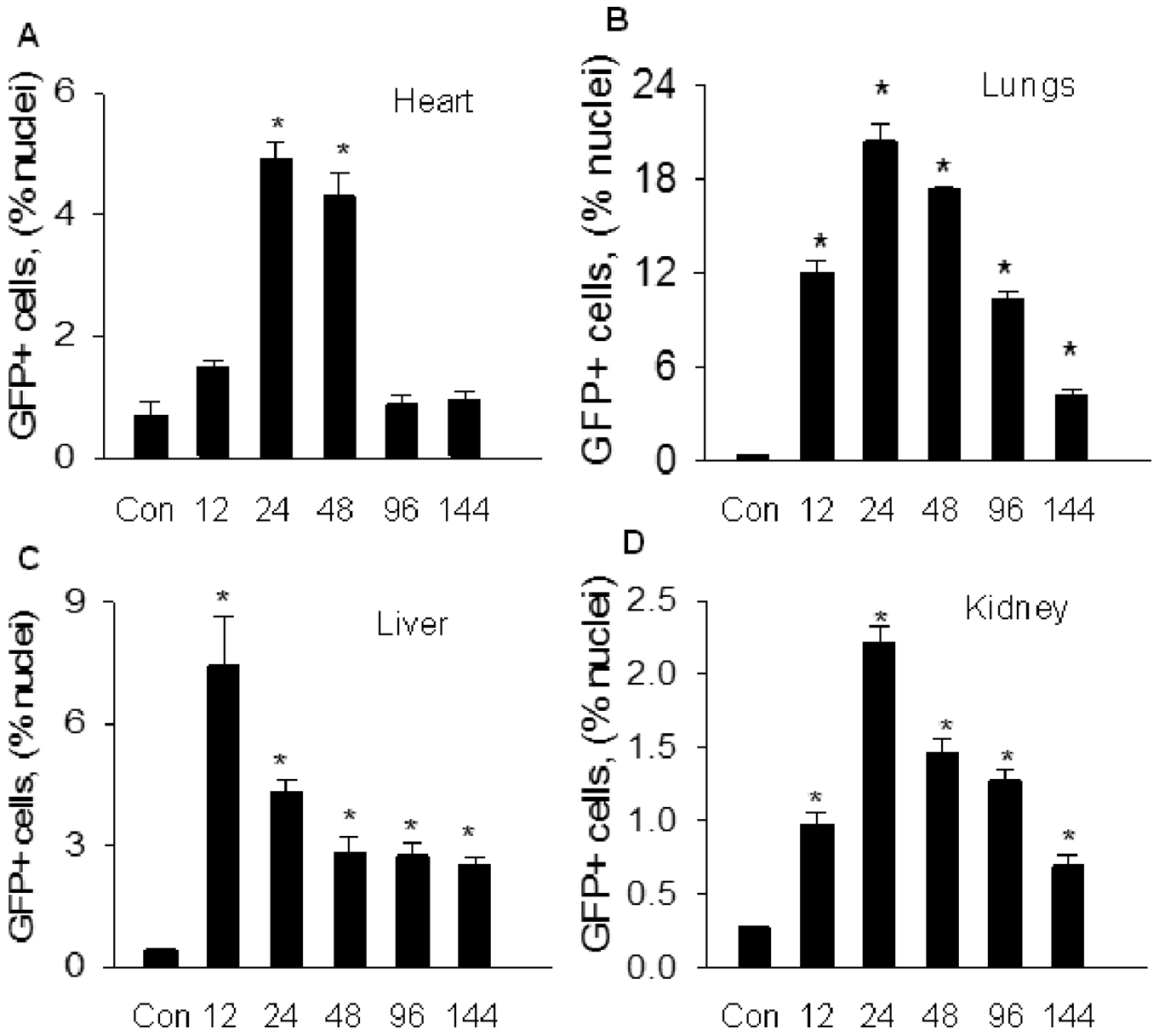
Time course profiles of BMDEPC recruitment into multiple organs. WT-GFP-BM mice were injected with saline (Con) or LPS (2 mg/kg, i.p.), and organs harvested at 12, 24, 48, 96 and 144 hours after LPS injection. Cryosections were prepared and stained with green fluorescence protein (GFP, genetic marker of BMDEPC) antibody and nuclei counterstained with DAPI (4, 6-diamidino-2-phenylindole). Number of GFP+ bone marrow derived endothelial progenitor cells (BMDEPCs) on each slide from heart (**A**), lungs (**B**), liver (**C**) and kidney (**D**) was counted and expressed as a percentage of total cells as revealed by DAPI nuclear staining. Mean ± SEM of 5 mice per group. *, p<0.05, compared with control group. There was an organ-dependent variation in numbers and kinetics of BMDEPC recruitment.

MOI was associated with a remarkably increased BMDEPCs recruitment. However, both the quantities and kinetics of BMDEPC recruitment varied significantly with organs (Fig. 1). The lung had the highest number of BMDEPC recruitment, but liver, heart and kidney had only 1/3, 1/4 and 1/9 of the number of BMDEPCs in lungs at the peak of BMDEPC recruitment (Fig. 1). Number of recruited BMDEPCs was in the order of lung > liver > heart > kidney. Kinetically, BMDEPCs entered into liver earlier. BMDEPC recruitment peaked at 12 hours in liver, but peaked at 24 hours post-LPS in heart, lungs and kidney (Fig. 1). On other hand, number of BMDEPCs decreased to control level by 96 hours in heart, but remained significantly higher than control in lungs, liver and kidney even at 144 hours post-LPS (Fig. 1).

**Figure 2:**
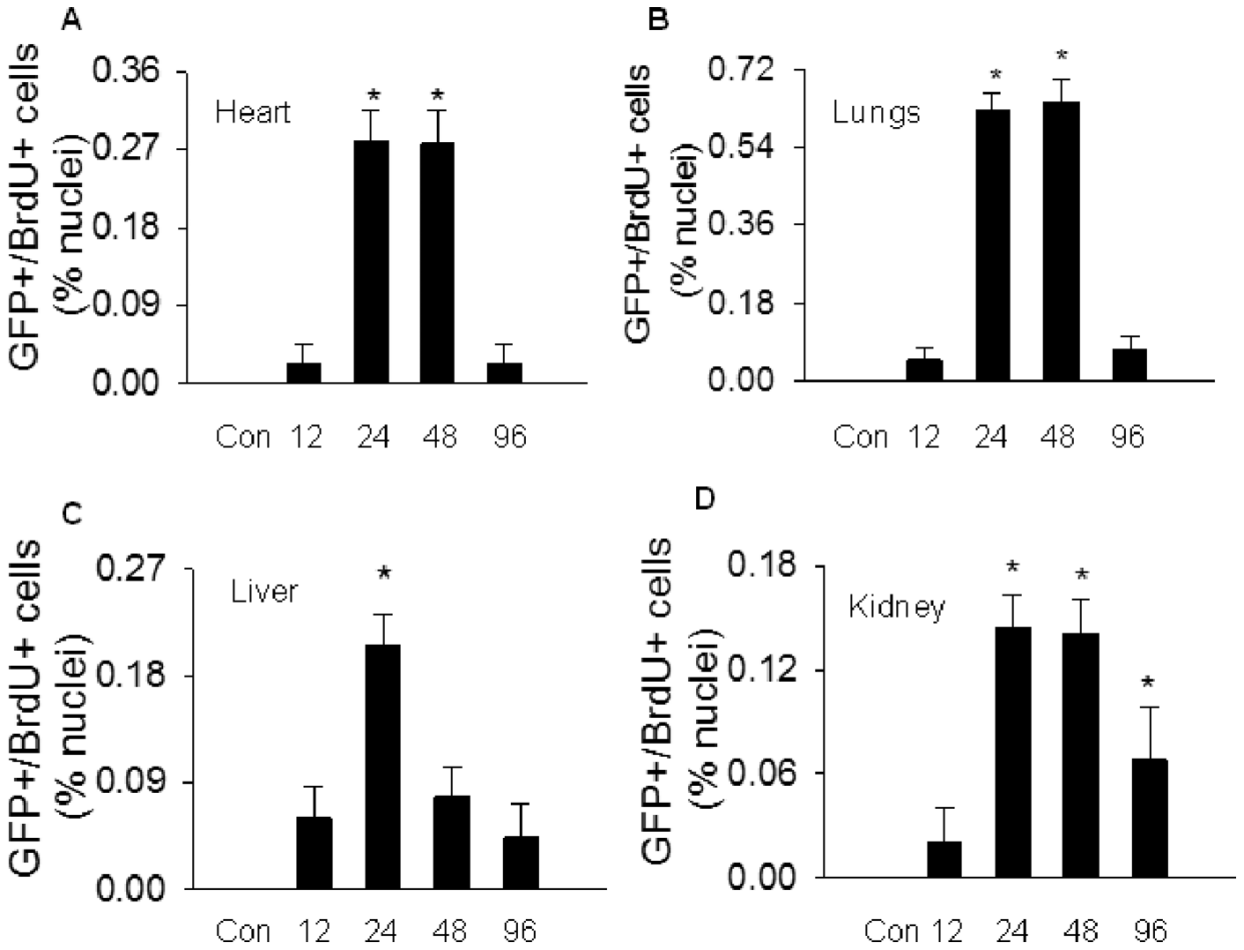
Characterization of BMDEPC proliferation in multiple organs. WT-GFP-BM mice were injected with saline (Con) or LPS and BrdU (100 μg/kg, i.v., to label proliferating cells). Organs were harvested at 12, 24, 48 or 96 hours after LPS and 4 hours after BrdU injection. Cryosections were prepared and stained with GFP plus BrdU antibodies to identify proliferating BMDEPCs, and nuclei counterstained with DAPI. Number of GFP+/BrdU+ proliferating BMDEPCs on each slide from heart (**A**), lung (**B**), liver (**C**) and kidney (**D**) was counted and expressed as a percentage of total cells as revealed by DAPI nuclear staining. Mean ± SEM of 5 mice per group. *, p<0.05, compared with control group. There was an organ-dependent variation in numbers and kinetics of BMDEPC proliferation.

Likewise, an organ-dependent variation in BMDEPC proliferation was observed. Number of proliferating BMDEPCs was 2-, 3- and 4-fold higher in lungs than in heart, liver and kidney at peak of BMDEPC proliferation. Number of proliferating BMDEPCs peaked at 24 hours in all 4 organs, and remained at the peak level at 48 hours post-LPS in heart, lungs and kidney, but not in liver. In liver, number of proliferating BMDEPCs has decreased to 42% of its peak level at 48 hours (Fig. 2). At 96 hours, number of proliferating BMDEPCs decreased to control level in heart, lungs and liver, but not in kidney (Fig. 2).

### P50 gene deletion inhibits LPS-induced NF-κB activity in chimeric mice

We inhibited NF-κB activity using p50-KO mice. We have previously demonstrated that LPS-induced NF-κB complex is composed predominantly of p50/p65 heterodimer with minimal p50/50 homodimers [37]. The lack of p50 protein in p50-KO mice diminishes or greatly reduces nuclear translocation of p50/p65 heterodimer, leading to an inhibition of NF-κB activation.

To examine the effects of systemic or compartment-selective blockade of NF-κB activity on BMDEPC recruitment and proliferation, we generated 4 chimeric mouse strains: WT-GFP-BM, p50-NG-BM, p50-GFP-BM and WT-NG-BM that carry an intact p50 gene or a disrupted p50 gene in all cell types, in stromal/parenchymal cells or in BM/blood cells, and overexpress GFP on BMDEPCs. A description of those mouse strains was given in Table S1. Two months after BM transplantation, we confirmed high degree of BM chimerism of the 4 chimeras by showing that BM cells of the chimeras were 92% to 95% of donor BM origin (Fig. S2).

**Figure 3:**
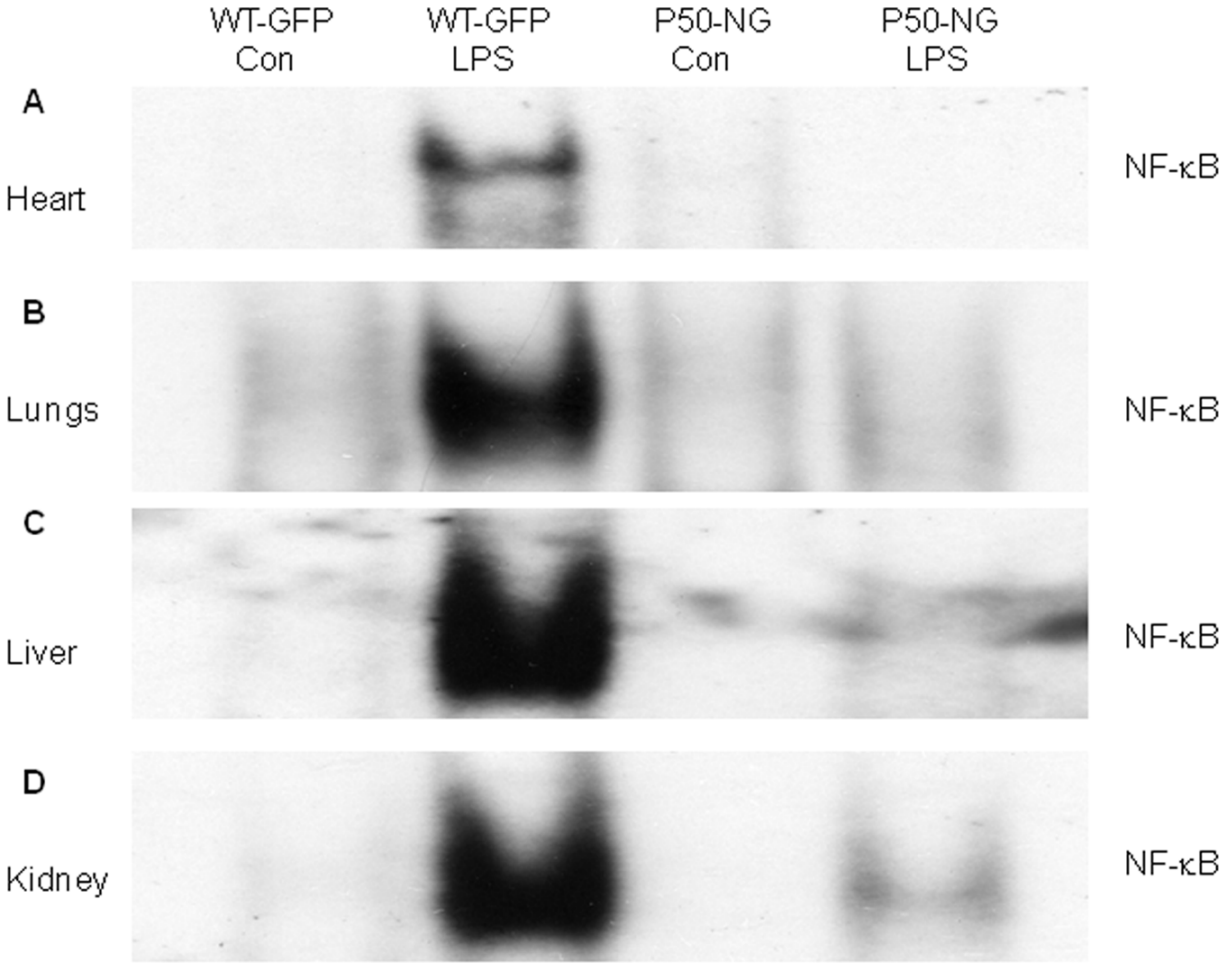
Electrophoretic mobility shift assay (EMSA) autoradiographs show that p50 gene deletion blocks LPS-induced NF-κB activation. WT-GFP-BM and p50-NG-BM mice were injected with saline (Con) or LPS, and organs harvested 6 hours post-LPS. Nuclear protein was extracted and NF-κB DNA binding activity in heart (**A**), lungs (**B**), liver (**C**) and kidney (**D**) was measured by EMSA using ^32^P-labeled NF-κB oligonucleotide probe. Representative of 3 independent experiments.

To confirm that p50 gene deletion blocks LPS-induced NF-κB activation in the chimeras, WT-GFP-BM and p50-NG-BM mice were injected with LPS and NF-κB DNA binding activity measured. EMSA showed that LPS induced a markedly increased NF-κB binding activity in heart, lungs, liver and kidney in WT-GFP-BM, which was diminished or greatly reduced in all 4 organs of p50-NG-BM mice (Fig. 3). This result confirms that p50 gene deletion blocks LPS-induced NF-κB activation in the chimeras.

### Effect of P50 gene deletion on BMDEPC recruitment

**Figure 4:**
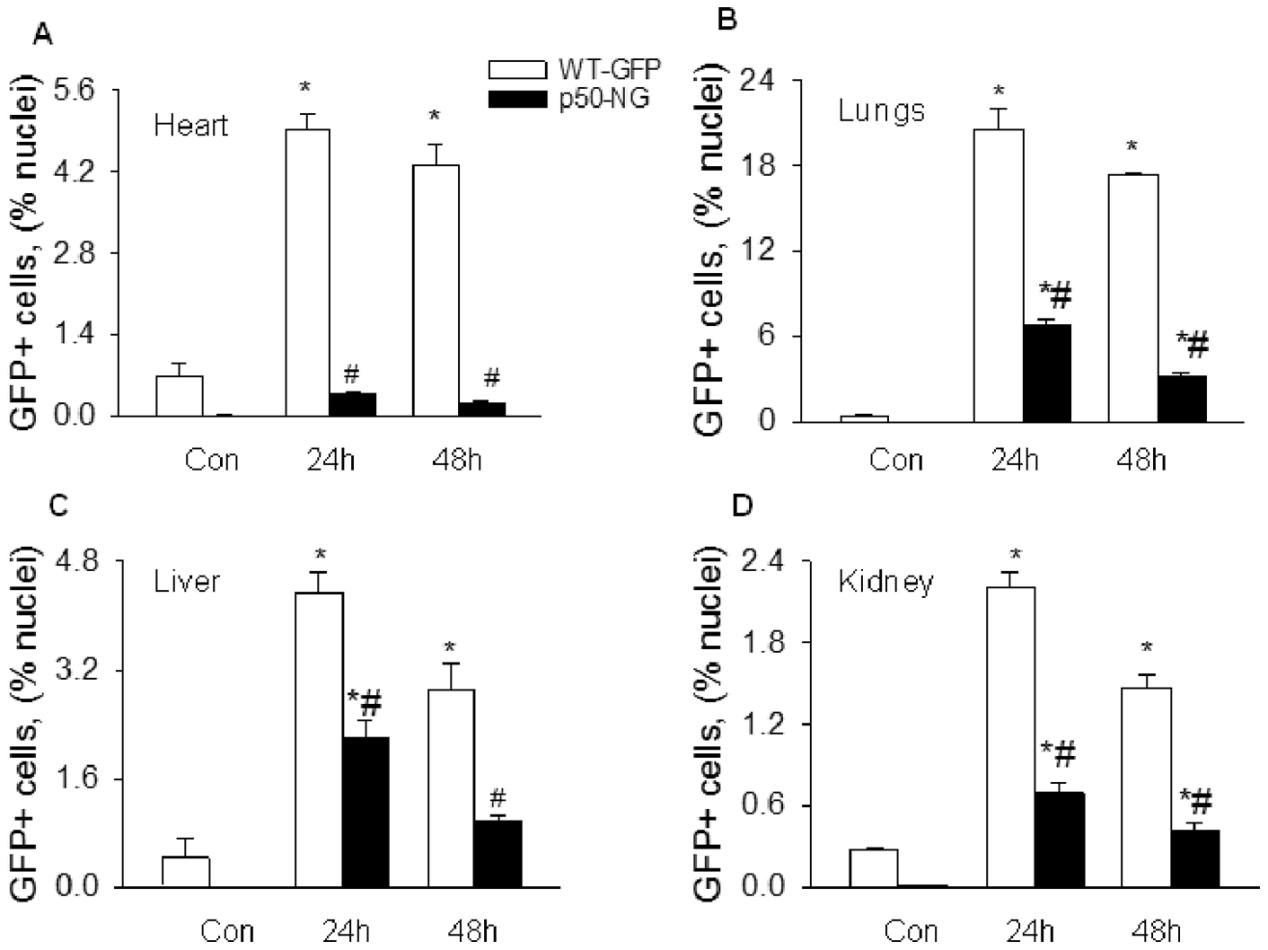
Systemic blockade of NF-κB activity inhibits BMDEPC recruitment in multiple organs. WT-GFP-BM and p50-NG-BM mice were injected with saline (Con) or LPS, and organs harvested at 24 and 48 hours post-LPS. Cryosections were prepared and stained with GFP antibody and nuclei counterstained with DAPI. Number of GFP+ BMDEPCs on each slide from heart (**A**), lung (**B**), liver (**C**) and kidney (**D**) was counted, expressed as a percentage of total cells as revealed by DAPI nuclear staining, and compared between WT-GFP-BM and p50-NG-BM mice. Mean ± SEM of 5 mice per group. *, p<0.05, compared with controls. #, p<0.05, compared with WT-GFP-BM mice at 24 or 48 hours.

We next examined the effect of p50 gene deletion on LPS-induced BMDEPC recruitment using WT-GFP-BM and p50-NG-BM mice (Table S1). Cryosections of heart, lungs, liver and kidney were prepared at 24 and 48 hours after saline or LPS injection, and stained with anti-GFP antibody. GFP+ BMDEPCs were counted and expressed as a percentage of total cells as revealed by DAPI nuclear staining. Number of BMDEPCs increased markedly in all 4 organs of WT-GFP-BM mice at 24 and 48 hours post-LPS (Fig. 4). LPS-induced BMDEPC recruitment was reduced by 95%, 81% and 72% at 48 hours post-LPS in heart, lungs and kidney of p50-NG-BM mice, whose p50 gene was deleted in all cells (Fig. 4). This result indicates an obligatory role for NF-κB in mediating LPS-induced BMDEPC recruitment into multiple organs.

**Figure 5:**
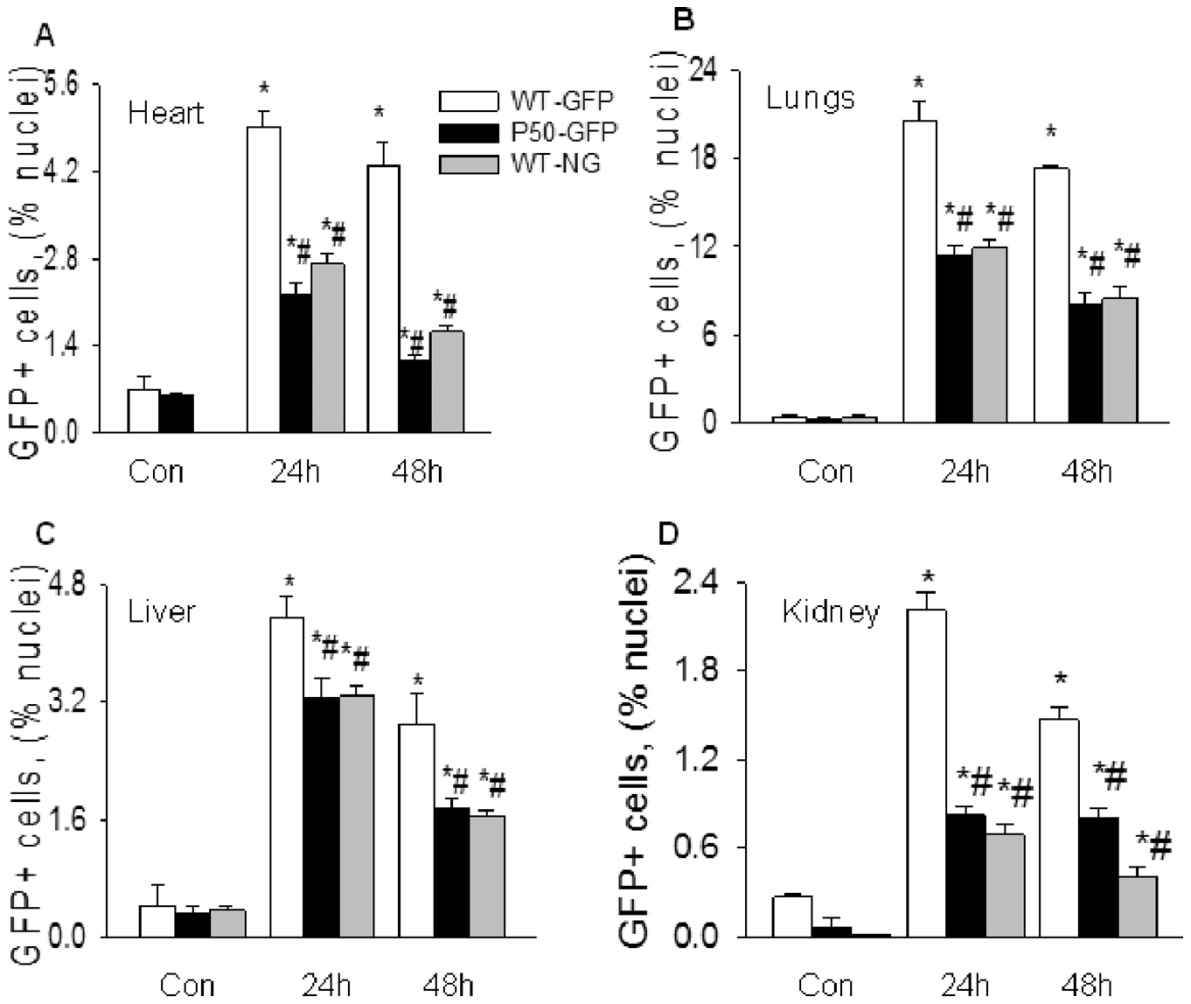
Selective blockade of stromal/parenchymal cell or BM/blood cell intrinsic NF-κB activity similarly inhibits BMDEPC recruitment. WT-GFP-BM, p50-GFP-BM and WT-NG-BM mice were injected with saline (Con) or LPS, and organs were harvested at 24 and 48 hours post-LPS. Cryosections were prepared and stained with GFP antibody and nuclei counterstained with DAPI. Number of GFP+ BMDEPCs on each slide from heart (**A**), lung (**B**), liver (**C**) and kidney (**D**) was counted, expressed as a percentage of total cells as revealed by DAPI nuclear staining, and compared between WT-GFP-BM and p50-GFP-BM, or WT-NG-BM mice. Mean ± SEM of 5 mice per group. *, p<0.05, compared with controls. #, p<0.05, compared with WT-GFP-BM mice at 24 or 48 hours. Blockade of intrinsic NF-κB activity in stromal/parenchymal cells (black bars) or in BM/blood cells (gray bars) similarly inhibits BMDEPC recruitment.

Using WT-NG-BM and p50-GFP-BM mice (Table S1), we deciphered the roles of BM/blood cell and stromal/parenchymal cell intrinsic NF-κB activities in regulating BMDEPC recruitment. Selective deletion of p50 gene in BM/blood cells (WT-NG-BM) or in stromal/parenchymal cells (p50-GFP-BM) reduced the number of recruited BMDEPCs by a similar extent (Fig. 5 gray and black bars), suggesting that NF-κB activities in the two compartments contribute equally to the regulation of LPS-induced BMDEPC recruitment.

The levels of inhibition of LPS-induced BMDEPC recruitment achieved by p50 gene deletion, either universal or compartment-selective, varied significantly between organs. Among the 4 organs, p50 gene deletion caused the highest inhibition in heart and the lowest inhibition in liver. The % inhibition of BMDEPC recruitment by universal p50 gene deletion was heart > lungs > kidney > liver (Figs. 4 and 5).

### Effect of P50 gene deletion on BMDEPC proliferation

Proliferation is an important mechanism by which BMDEPCs mediate endothelial and organ repair. We examined the role of NF-κB in regulating BMDEPC proliferation. The chimeric mice were injected with saline or LPS, and then with bromodeoxyuridine (BrdU) to label proliferating cells *in vivo* 4 hours prior to tissue collection. Cryosections from heart, lungs, liver and kidney were prepared and stained with anti-GFP plus anti-BrdU antibodies. GFP+/BrdU+ proliferating BMDEPCs were counted and expressed as a percentage of total cells as revealed by DAPI nuclear staining.

**Figure 6:**
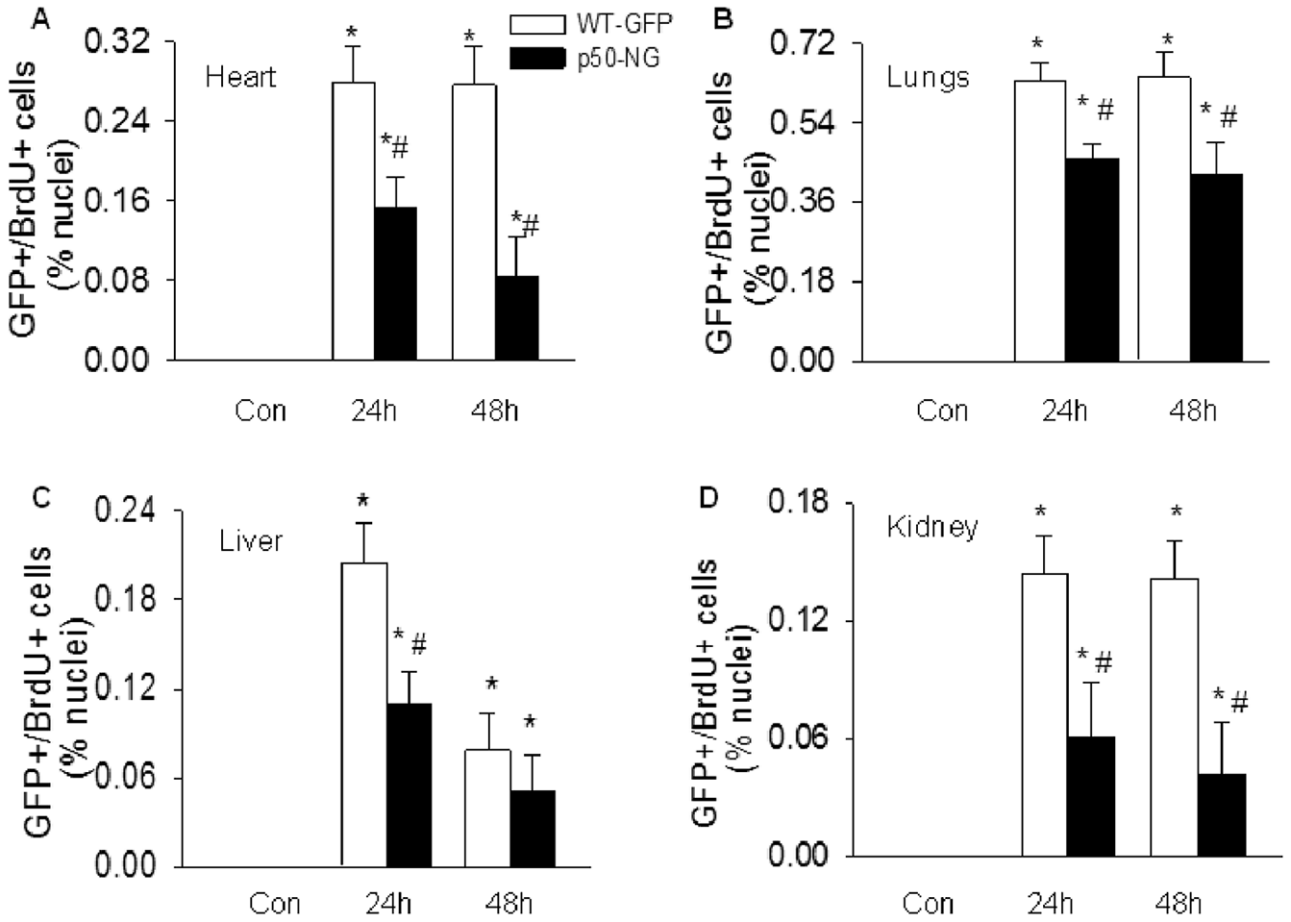
Systemic blockade of NF-κB activity inhibits BMDEPC proliferation in multiple organs. WT-GFP-BM and p50-NG-BM mice were injected with saline (Con) or LPS and BrdU (to label proliferating cells). Organs were harvested at 24 and 48 hours after LPS and 4 hours after BrdU injection. Cryosections were prepared and stained with GFP plus BrdU antibodies to identify proliferating BMDEPCs, and nuclei counterstained with DAPI. Number of GFP+/BrdU+ proliferating BMDEPCs on each slide from heart (**A**), lung (**B**), liver (**C**) and kidney (**D**) was counted, expressed as a percentage of total cells as revealed by DAPI nuclear staining, and compared between WT-GFP-BM and p50-NG-BM mice. Mean ± SEM of 5 mice per group. *, p<0.05, compared with controls. #, p<0.05, compared with WT-GFP-BM mice at 24 or 48 hours.

LPS caused a marked increase in number of proliferating BMDEPCs in all 4 organs of WT-GFP mice. Systemic NF-κB blockade by universal p50 gene deletion (p50-NG mice) significantly reduced number of proliferating BMDEPCs in all 4 organs (Fig. 6). However, the levels of inhibition varied significantly among organs. At 24 and 48 hours post-LPS, universal p50 gene deletion inhibited LPS-induced BMDEPC proliferation by 58% and 70% in kidney, but only by 27% and 34% in lungs (Fig. 6). At 48 hours post-LPS, universal p50 gene deletion did not significantly inhibit LPS-induced BMDEPC proliferation in liver (Fig. 6). The % inhibition of BMDEPC proliferation by universal p50 gene deletion was kidney > heart > liver > lung.

**Figure 7:**
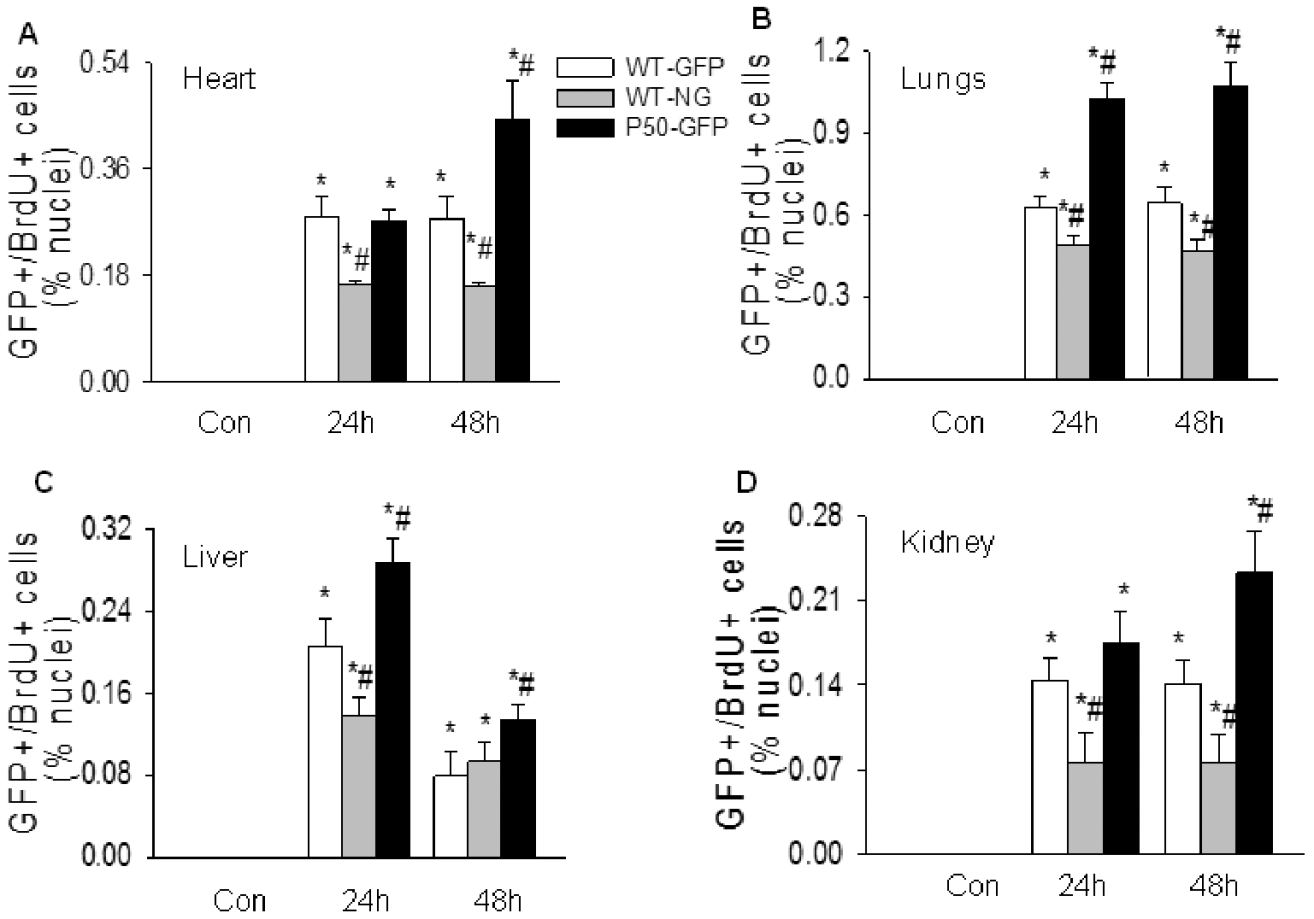
Selective blockade of BM/blood cell or stromal/parenchymal cell intrinsic NF-κB activity has opposite effects on BMDEPC proliferation. WT-GFP-BM, WT-NG-BM and p50-GFP-BM mice were injected with saline (Con) or LPS and BrdU (to label proliferating cells). Organs were harvested at 24 and 48 hours after LPS and 4 hours after BrdU injection. Cryosections were prepared and stained with GFP plus BrdU antibodies to identify proliferating BMDEPCs, and nuclei counterstained with DAPI. Number of GFP+/BrdU+ proliferating BMDEPCs on each slide from heart (**A**), lung (**B**), liver (**C**) and kidney (**D**) was counted, expressed as a percentage of total cells as revealed by DAPI nuclear staining, and compared between WT-GFP-BM and WT-NG-BM or p50-GFP-BM mice. Mean ± SEM of 5 mice per group. *, p<0.05, compared with controls. #, p<0.05, compared with WT-GFP-BM mice at 24 or 48 hours. Blockade of intrinsic NF-κB activity in BM/blood cells (gray bars) inhibits, but in stromal/parenchymal cells (black bars) augments BMDEPC proliferation.

BM/blood cell and stromal/parenchymal cell intrinsic NF-κB activities play opposite roles in regulating BMDEPC proliferation. Selective deletion of p50 gene in BM/blood cells (WT-NG mice) significantly inhibited, but in stromal/parenchymal cells (p50-GFP mice) significantly augmented LPS-induced BMDEPC proliferation in all 4 organs (Fig. 7). There appeared to be an organ-dependent variation in the inhibition or augmentation of BMDEPC proliferation caused by selective deletion of p50 gene in BM/blood cells or in stromal/parenchymal cells (Figs. 7). At 24 and 48 hours post-LPS, selective p50 gene deletion in BM/blood cells inhibited LPS-induced BMDEPC proliferation by 47% and 46% in kidney, but only by 22% and 27% in lungs (Figs. 7B and 7D, gray bars). At these time points, selective p50 gene deletion in stromal/parenchymal cells augmented LPS-induced BMDEPC proliferation by 21% and 65% in kidney, but by 63% and 67% in lungs (Figs. 7B and 7D, black bars).

## Discussion

While whether BMDEPCs contribute to endothelial and organ repair remains a subject of intense debate, an increased recruitment of BM-derived EPCs/SPCs has been consistently demonstrated to have beneficial effects [5]–[24]. Enhancing BMDEPC recruitment could be useful therapeutic strategy for the treatment of MOI. Towards this end, we need to better understand the mechanisms and pathways regulating BMDEPC recruitment and proliferation following MOI. Elucidation of mechanisms and pathways mediating BMDEPC recruitment and proliferation in injured organs may also reveal microenvironmental factors that influence BMDEPC's function in the injured organs, which may provide important information guiding the development of new approaches to improve BMDEPC-based cell therapies. To our knowledge, this is the first study that characterized the kinetics of BMDEPC recruitment and proliferation, and defined the role of NF-κB in regulating BMDEPC recruitment and proliferation following septic MOI. We reported several findings that have not previously been reported, and demonstrated several interesting conclusions that may improve our understanding of the mechanisms of BMDEPC recruitment and proliferation following endotoxemic MOI.

First, both the quantities and kinetics of BMDEPC recruitment and proliferation are highly organ-dependent. Different organs of same animal had different numbers of BMDEPC recruitment. The difference was more than 9-fold between organs with the highest and lowest BMDEPC recruitment. Likewise, numbers of proliferating BMDEPCs differed markedly between organs of the same animal. The difference was more than 8-fold between organs with the highest and lowest BMDEPC proliferation. Kinetically, BMDEPC recruitment and proliferation in different organs followed different time courses. BMDEPC recruitment reached its peak at 12 hours post-LPS in liver, but at 24 hours in other 3 organs. On other hand, number of recruited BMDEPCs returned to control level in heart by 96 hours, but retained significantly higher than control in lungs, liver or kidney even 144 hours post-LPS. BMDEPC proliferation in different organs showed different time course profiles. At 48 hours post-LPS, number of proliferating BMDEPCs has decreased to less than half of its peak value in liver, while number of proliferating BMDEPCs remained at peak level in lungs, heart and kidney.

Second, NF-κB activity plays obligatory and organ-dependent roles in regulating BMDEPC recruitment and proliferation. Systemic blockade of NF-κB activity by universal p50 gene deletion markedly reduced numbers of recruited and proliferating BMDEPCs in all organs studied, and the level of inhibition was impressively high. At 48 hours post-LPS, universal p50 gene deletion inhibited LPS-induced BMDEPC recruitment and proliferation by 95% and 69% in heart, and by 72% and 70% in kidney, suggesting that NF-κB plays obligatory roles in mediating BMDEPC recruitment and proliferation. However, NF-κB appears to play complex and organ-dependent roles. The extent of inhibition of BMDEPC recruitment and proliferation by p50 gene deletion varied significantly with organs. At 24 hours post-LPS, universal p50 gene deletion inhibited LPS-induced BMDEPC recruitment by 92% in heart, but only by 49% in liver, and inhibited LPS-induced BMDEPC proliferation by 58% in kidney, but only by 27% in lungs.

NF-κB plays a complex role. Inhibition of BMDEPC proliferation was proportional to the inhibition of BMDEPC recruitment in one organ, but was not proportional in another organ. At 48 hours post-LPS, universal p50 gene deletion inhibited LPS-induced BMDEPC proliferation and recruitment by 70% and 72% in kidney, but inhibited BMDEPC proliferation by 34% and recruitment by 81% in lung, and inhibited proliferation by 35% and recruitment by 66% in liver. This result suggests that NF-κB plays complex roles in regulating BMDEPC proliferation and recruitment.

Third, NF-κB activities in different tissue compartments play distinct roles in regulating BMDEPC recruitment and proliferation. Both stromal/parenchymal and BM/blood cell intrinsic NF-κB activities mediate BMDEPC recruitment. NF-κB activities in the two compartments are synergistic in mediating BMDEPC recruitment. Blockade of NF-κB activities in both compartments by universal p50 gene deletion led to a significantly greater inhibition of BMDEPC recruitment than blockade of NF-κB activity in stromal/parenchymal cells or in BM/blood cells alone. By contrast, BM/blood cell intrinsic NF-κB activity mediates, but stromal/parenchymal cell intrinsic NF-κB activity inhibits LPS-induced BMDEPC proliferation. NF-κB activities in the two compartments play antagonistic roles in regulating BMDEPC proliferation.

A question raised is that if NF-κB activities in stromal/parenchymal and BM/blood cells played opposite roles in regulating BMDEPC proliferation, why blockade of NF-κB activities in both compartments by universal p50 gene deletion inhibited BMDEPC proliferation? One possible explanation is that the weak augmenting effect on BMDEPC proliferation was masked by the strong inhibitory effect on BMDEPC recruitment. Universal p50 gene deletion blocked stromal/parenchymal and BM cell/blood cell NF-κB activities simultaneously. The stimulating and inhibiting effects on BMDEPC proliferation caused by blocking stromal/parenchymal and BM/blood cell intrinsic NF-κB activities were balanced. At the same time, blockade of NF-κB activities in both compartments greatly reduced BMDEPC recruitment. For example, numbers of recruited BMDEPCs were reduced by 92% and 95% in the heart at 24 and 48 hours post-LPS. As a consequence, the number of proliferating BMDEPCs was significantly reduced by universal p50 gene deletion.

We observed that BMDEPCs were differentially recruited into lungs, heart, liver and kidney. Mechanisms underlying the different organ distribution of mobilized BMDEPCs are unclear, but suggested that the “sieve” action of pulmonary capillary beds may contribute to the high number of BMDEPC retention in lungs. Trapping of substantial numbers of BMDEPCs in lung capillary beds could reduce the number of BMDEPCs available for distribution to downstream organs. However, this interpretation cannot explain the differences between other organs. Other factors must be considered. Differences in microenvironmental factors and extent of injuries between organs may be important factors that are worth considering. It was reported in an unilateral kidney injury mouse model that EPCs were predominantly recruited into the injured kidney, but not the contralateral control kidney (2), indicating that different extent of injuries between organs can affect BMDEPC recruitment.

The organ-dependent variation in kinetics and NF-κB-mediated regulation of BMDEPC recruitment and proliferation suggests that different types of organ injuries may stimulate BMDEPC recruitment and proliferation via distinct mechanisms. Elucidation of the mechanisms underlying the differential BMDEPC recruitment and proliferation in different organs, and the organ-dependent variation in NF-κB-mediated regulation of BMDEPC recruitment and proliferation will greatly improve our understanding of the mechanisms of BMDEPC recruitment and proliferation following septic MOI.

Depending on models, context, stimuli and partner protein with which p50 interacts, NF-κB p50 has both pro- and anti-inflammatory effects [29], [36], [37], [43], [46], so does p50 gene deletion. P50 forms p50/p50/Bcl-3 (B-cell lymphoma 3-encoded protein) or p50/p50/HDAC-1 (histone deacetylase-1) repressor complex, which suppresses transcriptional expression of inflammatory genes [43], [44]. Under these circumstances, p50 gene deletion would augment inflammation and exacerbate tissue injury. On other hand, p50 form p50/p65 heterodimer, which mediates the transcriptional expression of a large number of inflammatory genes [29]. Under these conditions, p50 gene deletion would inhibit inflammation and attenuate tissue injury [29], [36], [37], [45], [46]. We suggest that p50 gene deletion here inhibits BMDEPC recruitment and proliferation by blocking the activation of the canonical NF-κB pathway, which is characterized by I-κBα degradation and nuclear translocation of p50/p65 heterodimer [27]–[29]. We have previously demonstrated that I-κBα degradation was a prerequisite for LPS-induced NF-κB activation [29], [37] and that I-κBα overexpression blocked LPS-induced NF-κB activation [34]. Our supershift assay revealed that LPS-induced NF-κB complex is composed predominantly of p50/p65 heterodimer, and contains minimal p50/p50 homodimer [37]. We demonstrated here that p50 gene deletion diminished or greatly reduced LPS-induced NF-κB band in all 4 organs of the chimeric mice. It is likely that the lack of p50 protein in p50-KO mice diminishes nuclear translocation of p50/p65 heterodimer, leading to an inhibition of NF-κB activation, which inhibits BMDEPC recruitment and proliferation. Our data adds a new role to the long list of functions that NF-κB plays.

In summary, we have created 4 chimeric mouse strains. Using these mouse models, we demonstrated that BMDEPC recruitment and proliferation displayed different characteristics and kinetics in different organs following endotoxemic MOI. NF-κB plays obligatory and organ-dependent roles in regulating BMDEPC recruitment and proliferation. NF-κB activities in different tissue compartments play distinct roles in the regulations. Both stromal/parenchymal and BM/blood cell intrinsic NF-κB activities mediate BMDEPC recruitment. By contrast, BM/blood cell intrinsic NF-κB activity mediates, but stromal/parenchymal cell intrinsic NF-κB activity inhibits BMDEPC proliferation. Our data provides new insights into the mechanisms regulating BMDEPC recruitment and proliferation following endotoxemc MOI.

## Supporting Information

Figure S1 **Low-dose LPS causes organ inflammation and injury in multiple organs.** Control and LPS groups of wild type mice were injected with saline or LPS (2 mg/kg, i.p.). At 24 hours after LPS injection, organ histology was evaluated, and tissue levels of IL-6 and myeloperoxidase (MPO) activity measured. **A**. Representative photographs of H&E staining of lung, kidney and liver sections show that LPS causes organ injury. Sections from the 3 organs of LPS-challenged mice exhibited increased inflammatory cell infiltration, alveolar, kidney sinusoidal and liver central vein congestion, and scattered areas of hemorrhage. Lung section showed an increased alveolar wall thickness and alveolar collapse. Kidney section showed signs of tubular swelling and cell injury. Liver section showed signs of hepatic cell injury. Scale bar, 50 µm. **B**. Bar graphs show that LPS causes marked increase in tissue levels of IL-6 in lung, kidney and liver. Mean ± S.M.E. of 5 mice per group. *: p<0.05, compared to control group. **C**. Bar graphs show that LPS markedly increases tissue levels of MPO activity in lung, kidney and liver. Mean ± S.M.E. of 5 mice per group. *: p<0.05, compared to control group.
(PDF)

Figure S2 **FACS analysis of BM chimerism.** Two months after BM transplant, BM mononuclear cells (BMMNCs) were isolated from donors, recipients and chimeras, stained with CD31 plus GFP antibodies, and analyzed. The degree of BM chimerism was evaluated based on percentage of donor-derived CD31+/GFP+ endothelial progenitor cells (EPCs) in BMMNC population of the chimera. **A**. Representative FACS pictures show percentage of CD31+/GFP+ EPCs (upper right quadrant) in BMMNCs from Tie2-GFP (donor), WT-GFP-BM (chimera) and WT (recipient) mice, demonstrating high degree of BM chimerism. **B**. Representative FACS pictures show percentage of CD31+/GFP+ EPCs (upper right quadrant) in BMMNCs of Tie2-GFP (donor), p50-GFP-BM (chimera) and NF-κB p50 gene knock out (p50-KO, recipient) mice, demonstrating high degree of BM chimerism. **C**. Representative FACS pictures show percentage of CD31+/GFP+ EPCs (upper right quadrant) in BMMNCs of NG (p50-KO-GFP, donor), WT-NG-BM (chimera), and WT(recipient) mice, demonstrating high degree of donor BM chimerism. **D**. Representative FACS pictures show percentage of CD31+/GFP+ EPCs (upper right quadrant) in BMMNCs of NG (p50-KO-GFP, donor), p50-NG-BM (chimera), and p50-KO (recipient) mice, demonstrating high degree of donor BM chimerism.
(PDF)

Table S1 **Mouse strains used.**
(PDF)
