# Supplementary figures and images for: An Obligatory Role of NF-κB in Mediating Bone Marrow Derived Endothelial Progenitor Cell Recruitment and Proliferation Following Endotoxemic Multiple Organ Injury in Mice
Source: PLoS One. 2014 Oct 21;9(10):e111087. doi: 10.1371/journal.pone.0111087 (PMC4205081; doi:10.1371/journal.pone.0111087)

**Figure S1**

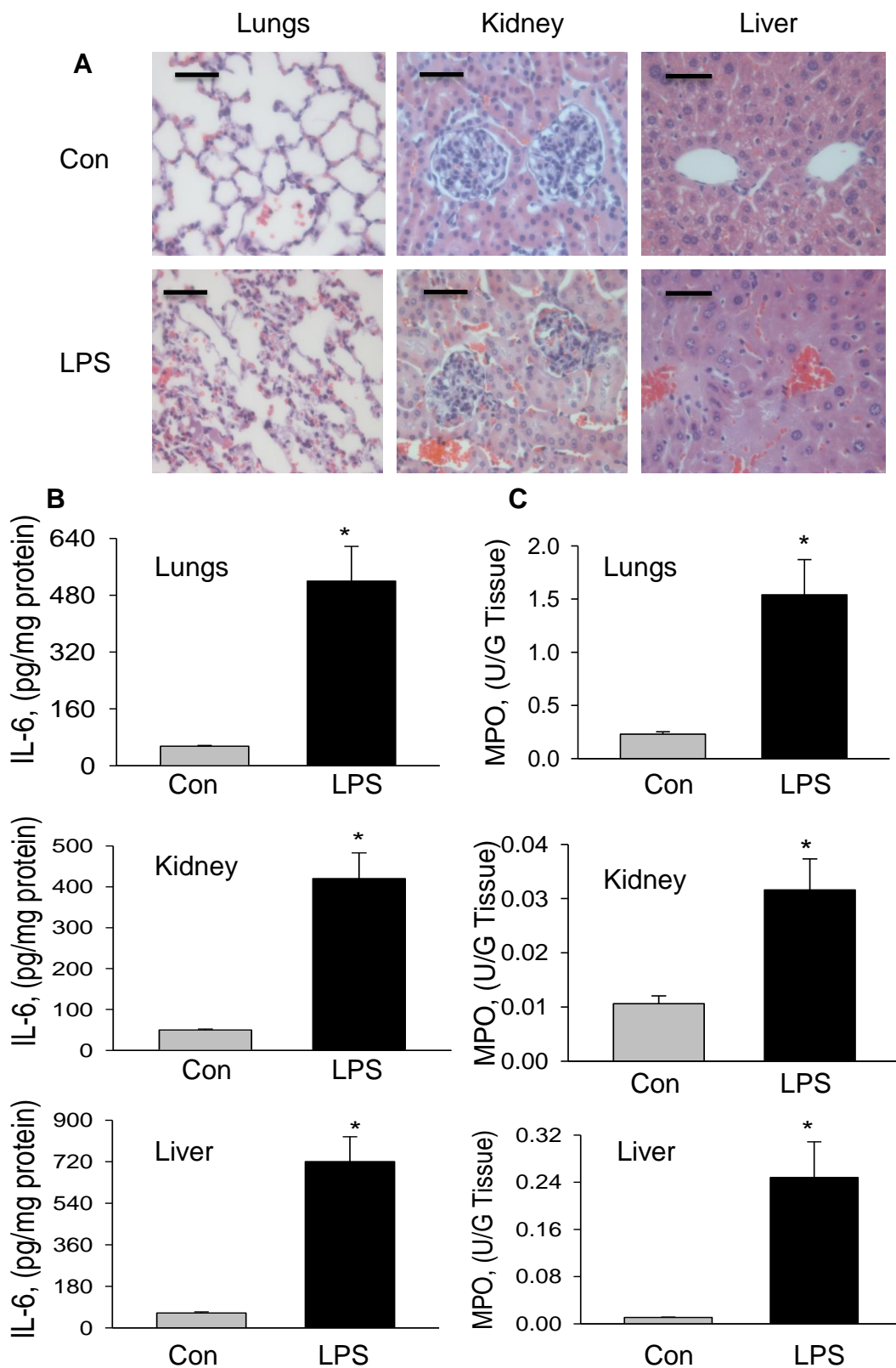

Supplement: Figure S1 — Low-dose LPS causes organ inflammation and injury in multiple organs. Control and LPS groups of wild type mice were injected with saline or LPS (2 mg/kg, i.p.). At 24 hours after LPS injection, organ histology was evaluated, and tissue levels of IL-6 and myeloperoxidase (MPO) activity measured. A. Representative photographs of H&E staining of lung, kidney and liver sections show that LPS causes organ injury. Sections from the 3 organs of LPS-challenged mice exhibited increased inflammatory cell infiltration, alveolar, kidney sinusoidal and liver central vein congestion, and scattered areas of hemorrhage. Lung section showed an increased alveolar wall thickness and alveolar collapse. Kidney section showed signs of tubular swelling and cell injury. Liver section showed signs of hepatic cell injury. Scale bar, 50 µm. B. Bar graphs show that LPS causes marked increase in tissue levels of IL-6 in lung, kidney and liver. Mean ± S.M.E. of 5 mice per group. *: p<0.05, compared to control group. C. Bar graphs show that LPS markedly increases tissue levels of MPO activity in lung, kidney and liver. Mean ± S.M.E. of 5 mice per group. *: p<0.05, compared to control group. (PDF) [file pone.0111087.s001.pdf]

**Figure S2**

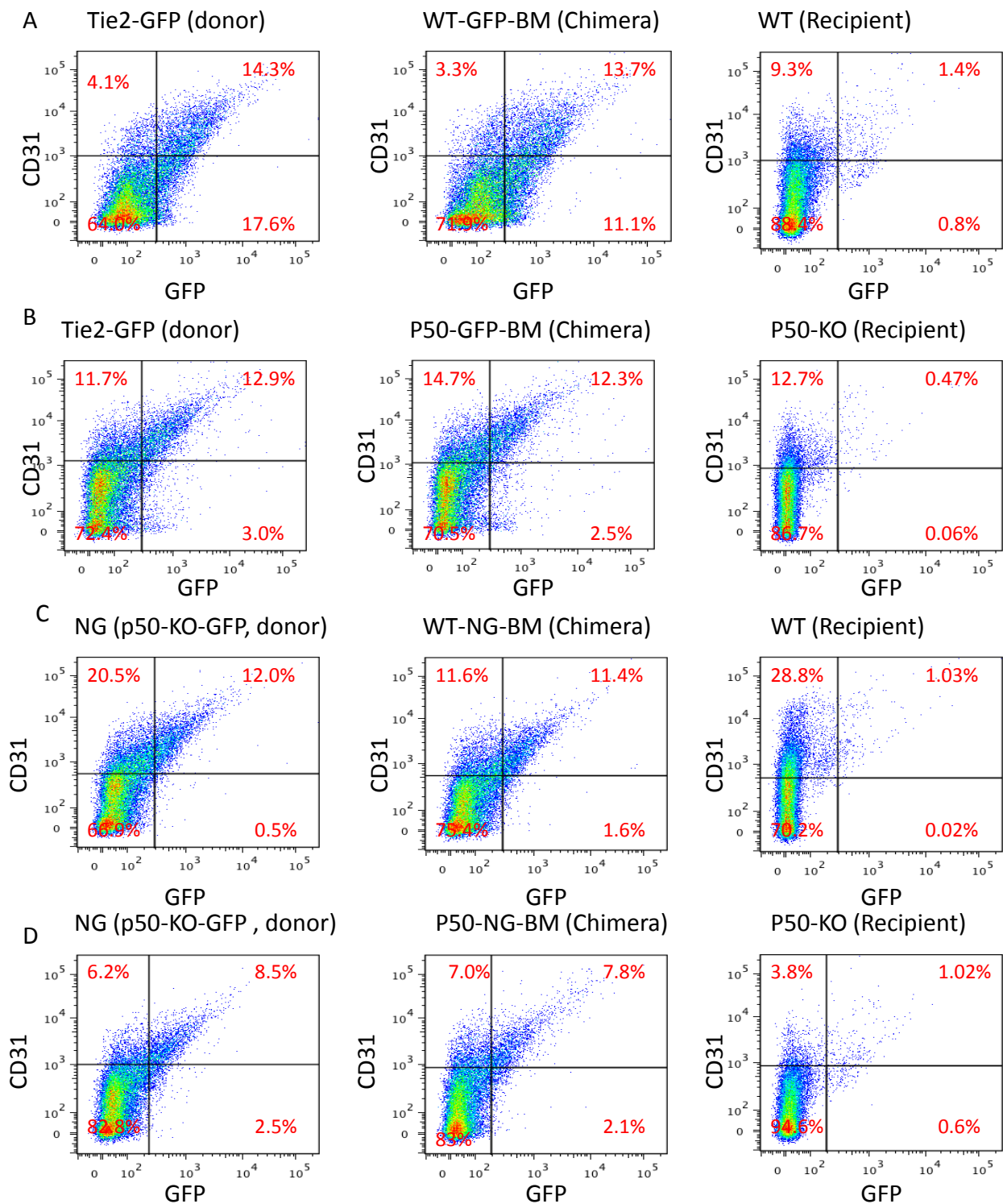

Supplement: Figure S2 — FACS analysis of BM chimerism. Two months after BM transplant, BM mononuclear cells (BMMNCs) were isolated from donors, recipients and chimeras, stained with CD31 plus GFP antibodies, and analyzed. The degree of BM chimerism was evaluated based on percentage of donor-derived CD31+/GFP+ endothelial progenitor cells (EPCs) in BMMNC population of the chimera. A. Representative FACS pictures show percentage of CD31+/GFP+ EPCs (upper right quadrant) in BMMNCs from Tie2-GFP (donor), WT-GFP-BM (chimera) and WT (recipient) mice, demonstrating high degree of BM chimerism. B. Representative FACS pictures show percentage of CD31+/GFP+ EPCs (upper right quadrant) in BMMNCs of Tie2-GFP (donor), p50-GFP-BM (chimera) and NF-κB p50 gene knock out (p50-KO, recipient) mice, demonstrating high degree of BM chimerism. C. Representative FACS pictures show percentage of CD31+/GFP+ EPCs (upper right quadrant) in BMMNCs of NG (p50-KO-GFP, donor), WT-NG-BM (chimera), and WT(recipient) mice, demonstrating high degree of donor BM chimerism. D. Representative FACS pictures show percentage of CD31+/GFP+ EPCs (upper right quadrant) in BMMNCs of NG (p50-KO-GFP, donor), p50-NG-BM (chimera), and p50-KO (recipient) mice, demonstrating high degree of donor BM chimerism. (PDF) [file pone.0111087.s002.pdf]
