# Supplementary material for: An Obligatory Role of NF-κB in Mediating Bone Marrow Derived Endothelial Progenitor Cell Recruitment and Proliferation Following Endotoxemic Multiple Organ Injury in Mice
Source: PLoS One. 2014 Oct 21;9(10):e111087. doi: 10.1371/journal.pone.0111087 (PMC4205081; doi:10.1371/journal.pone.0111087)
Supplement: Table S1 — Mouse strains used. (PDF) [file pone.0111087.s003.pdf]

Online Support Information

Table S1. Mouse strains used

Strains	Characteristics
P50-KO	Mice deficient in NF-κB p50 gene
Tie2-GFP	Mice overexpressing Tie2-GFP on endothelial lineage cells
NG	Mice deficient in p50 gene in all cell types and overexpressing GFP on endothelial lineage cells
WT-GFP-BM	Mice with an intact p50 gene and overexpressing GFP on BMDEPCs.
P50-NG-BM	Mice deficient in p50 gene in all cells and overexpressing GFP on BMDEPCs.
P50-GFP-BM	Mice deficient in p50 gene in stromal/parenchymal cells and overexpressing GFP on BMDEPCs.
WT-NG-BM	Mice deficient in p50 gene in bone marrow/blood cells and overexpressing GFP on BMDEPCs.
